# Using total quality management approach to improve patient safety by preventing medication error incidences^**^

**DOI:** 10.1186/s12913-017-2531-6

**Published:** 2017-09-04

**Authors:** Nadin Yousef, Farah Yousef

**Affiliations:** 1 Researcher in Pharmaceutical Sciences and Medical Researches, Lattakia, Syria; 2 Ph.D. candidate in Pharmaceutical Sciences, Damascus University, Damascus, Syria

**Keywords:** Six sigma approach, DAMIC process, Medication errors

## Abstract

**Background:**

Whereas one of the predominant causes of medication errors is a drug administration error, a previous study related to our investigations and reviews estimated that the incidences of medication errors constituted 6.7 out of 100 administrated medication doses. Therefore, we aimed by using six sigma approach to propose a way that reduces these errors to become less than 1 out of 100 administrated medication doses by improving healthcare professional education and clearer handwritten prescriptions.

**Methods:**

The study was held in a General Government Hospital. First, we systematically studied the current medication use process. Second, we used six sigma approach by utilizing the five-step DMAIC process (Define, Measure, Analyze, Implement, Control) to find out the real reasons behind such errors. This was to figure out a useful solution to avoid medication error incidences in daily healthcare professional practice. Data sheet was used in Data tool and Pareto diagrams were used in Analyzing tool.

**Results:**

In our investigation, we reached out the real cause behind administrated medication errors. As Pareto diagrams used in our study showed that the fault percentage in administrated phase was 24.8%, while the percentage of errors related to prescribing phase was 42.8%, 1.7 folds. This means that the mistakes in prescribing phase, especially because of the poor handwritten prescriptions whose percentage in this phase was 17.6%, are responsible for the consequent) mistakes in this treatment process later on. Therefore, we proposed in this study an effective low cost strategy based on the behavior of healthcare workers as **Guideline Recommendations** to be followed by the physicians. This method can be a prior caution to decrease errors in prescribing phase which may lead to decrease the administrated medication error incidences to less than 1%.

**Conclusion:**

This improvement way of behavior can be efficient to improve hand written prescriptions and decrease the consequent errors related to administrated medication doses to less than the global standard; as a result, it enhances patient safety. However, we hope other studies will be made later in hospitals to practically evaluate how much effective our proposed systematic strategy really is in comparison with other suggested remedies in this field.

**Electronic supplementary material:**

The online version of this article (doi:10.1186/s12913-017-2531-6) contains supplementary material, which is available to authorized users.

## Background

### Problem description

Medication errors are the most common type of medical errors in healthcare sectors. They may cause or lead to inappropriate medication use or patient harm [1]. 6–7% of hospital admissions are due to medication errors [2].

### Available knowledge

Medication error is a harmful event that may occur in different stages of patient treatment process. Hence, which step caused the problem cannot be well determined. However, medication errors can generally be classified either according to the stage of their occurrence (i.e. prescribing, drug administering, dispensing or error monitoring) or according to their damage intensity [3].

World Health Organization summarized in its recent report related to medication errors the key factors that may lead to the occurrence of these errors. These factors can be attributed to health care professionals, patients, work environment, medicines, tasks, computerized information systems, or primary-secondary care interface issues [3].

Different approaches were proposed to solve this problem. One of them is restoring to automated data systems, medication reviews and reconciliation, educating health care workers, and identifying Multicomponent interventions [4–8].

### Rational and specific aims

Total Quality Management (TQM), is a methodology of management for continuously improving the quality of products and processes to meet or exceed customer expectations [1], and; on the other hand, Six Sigma is a business management strategy which seeks to improve the quality of process outputs. This business management depends on using a set of tools which are known as DMAIC (Define, Measure, Analyze, Improve, and Control). Therefore, Integrating Six Sigma with TQM program improves the process through detailed data analysis, and it makes TQM efforts more successful [9]. In other words, the achievement of six sigma methodology applied in this research paper, integrated with TQM, was a trial to work on health care professional educating to prevent medication errors occurring in healthcare sectors. We aimed to reduce the medication error incidence to less than the global standards indication; 1 out of 100 administrated medication doses, within a period of 13 weeks with no high expenditure.

## Methods

A **medication error** is any harmful event that may cause or lead to an inappropriate medication use or patient harm whether such an error comes from the health care professional, the patient, or the consumer. These errors are typically considered to be related to administration of a medication. In fact, they may also include errors in ordering or delivering the medications [10].

We used the following definitions of medication errors according to the step of their occurrence:

### Prescribing errors

They occur as a result of a prescribing decision or prescription writing process [10]. It includes mistakes made by the physician when ordering a medication; incorrect drug selection, route, the frequent of administration,, dosage form, instructions for use of a drug product, wrong drug, drug to which patient is allergic, Drug-Drug Interactions (DDIs), bad Controlled Drug Substances (CDS), not following good CDS, and wrong patient errors [11].

### Administration errors

Such errors usually occur when deviating from the physician’s order according to what is written in the patient’s chart [12]. They errors include unlicensed drug, over dose, wrong dose, missing dose, wrong form of administration, wrong technique, wrong time [13].

### Dispensing errors

The deviations from the physician’s order, made by the pharmacy staff when distributing the medications to the nursing team or to the patients in an ambulatory setting [14]. The dispensing process is an integral part of the quality of medicine usage that forms together with patient counseling the core professional activities of a pharmacist. The process of dispensing and counseling is composed of a sequence of steps. If any of these steps has been interrupted or completed incorrectly, this could result in poor quality outcomes for the patient. This type of errors generally refers to errors in the dispensing process (wrong drug, wrong dose strength, incorrectly labeled directions, or drug dispensed to wrong patient). These errors cannot be detected or corrected prior to the patient leaving the pharmacy. This may lead; as a consequence, to less effective outcomes of treatment for the patient [15].

### Monitoring errors

They are the failure to review a prescribed therapeutic plan for appropriateness and detection of problems, or weakness to use appropriate clinical or laboratory data for adequate assessment of patient response to prescribed therapy [16].

### Interventions, measures and analysis

This project was conducted at a general governmental hospital. The number of beds was 93 beds, and the number of physicians was 137 physicians, and the number of nurses was 318 nurses. We systematically studied the process of medication application by the health care professionals in this hospital. Then, we introduced six sigma in our health care setup for the provision of the patient’s safety. Six Sigma’s approach of problem (Definition, measurement, and statistical analysis, improvement, and control plans) was involved in our study. The six sigma quality improvement team used the five-step DMAIC process for every project [17].

In other words, we formulated theories by brainstorming to figure out the real causes of medication errors after studying the applied treatment process. For that, Medication Error Causes- Data Sheet was used to determine the real cause behind each type of medication errors defined above and its percentage. This was based on nurses’ answers. Data-analysis tool was Pareto diagram.

Table 1 defines each phase of the DMAIC process (Define, Measure, Analyze, Implement, Control).

**Table 1:** Definitions for the DMAIC Process (Define, Measure, Analyze, Improve, Control)

Phase	Definition	Components
Define	Identify a project Establish the project	A. Identify the project. B. Identify the problem. C. Identify the objective.
Measure	Understand the current process in need of improvement	A. SIPOC (Suppliers, Inputs, Process, Outputs, Customers). B. Voice of the customer. C. Symptoms analyze: (Incidence of medication error): 1- Operational definition. 2- Define boundaries).
Analyze	Use statistical analysis to understand causes and effects in relation to the current process.	A. Formulate Theories & Cause-Effect Diagrams. B. Test Theories C. Data Collection. D. Identify Root Cause(s).
Improve	Develop a plan that can be validated by statistical data to improve the process	A. Evaluate alternatives. B. Design remedy & Design for culture. C. Prove effectiveness & Implement.
Control	Establish a monitoring tool or mechanisms to ensure that the process will be sustained	Design effective quality controls: A. Foolproof the improvement. B. Audit the controls.

## Results and discussion

### Definition phase

Medication errors are a global issue that causes harm and even death. They are very costly and adversely influence patients’ safety, nurses and organizations.

A previous study in our pilot investigations and reviews estimated that the incidence of medication errors was 6.7 out of 100 administrated medication doses, while the global standards indicated that the incidence of medication errors should not exceed 1 out of 100 administrated medication doses.

Our objective is to reduce the medication error incidence to less than 1 out of 100 administrated medication doses, within a period of 13 weeks with no high expenditure.

### Measurement phase

#### SIPOC

**Fig. 1:**
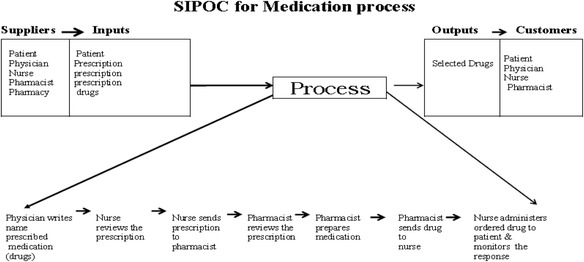
SIPOC for Medication process (Suppliers, Inputs, Process, Outputs, Customers)

**Suppliers** (Patient, Physician, Nurse, Pharmacist, Pharmacy), **Inputs** (Patient, Prescription, drugs), **Process** (Prescribing, Dispensing, Administrating, Monitoring), **Outputs** (Selected Drugs), **Customers** (Patient, Physician, Nurse, Pharmacist). Figure 1 shows SIPOC for medication process.

#### Voice of the customer

It includes four steps as shown in Table 2. Table 3 shows the customer feedback, Critical Customer Requirements (CCRs), Critical to Quality (CTQs), and Targets.

**Table 2:** Voice of the customer steps

Step number	Step title	Components
1	Develop a customer–Focused business strategy	- Assess the business needs. - Identify customer segments.
2	Listening to the VOC	- To obtain useful and valid customer information and feedback: - Select research methods to gather customer information. - Probe for complete understanding.
3	Translating voice of the customer (VOC) into critical customer requirements (CCRs).	- Organize and verify customer needs data into CCRs. - Determine CCR priorities. - Identify CCR measurement and target
4	Developing measures and indicators	- Translate the CCRs into output indicators: - Identify and select output indicators. - Establish output performance targets.

**Table 3:** Voice of customer

Voice of customer	CCRs (Critical Customer Requirements)	CTQs (Critical To Quality)	Targets
Physician’s writing on the doctor’s order form is difficult to read	Poor hand writing	Write orders legibly	Percentage of orders that are illegible is less than 15%
Nurses confuse between two drugs with similar names	Use Unapproved abbreviations	Write medication orders that can be accurately interpreted	Percentage of orders that contain “non-approved” abbreviations is less than 15%
Medication labels/packaging are damaged	Wrong drug	The Right Drug	Getting the Right Drug Every Time!
Medication is administered by a route that is different from the one ordered.	Wrong Route	The Right Route	Getting the Right Route Every Time!
Physician prescribes the wrong dose	Wrong dose	The Right Dose	Getting the Right Dose Every Time!
Nurse miscalculates the dose	Wrong dose error	The Right Dose	Getting the Right Dose Every Time!
A medication was given to the incorrect patient due to failure to properly identify patient or order.	wrong patient	Right patient	Getting the Right Patient Every Time!
The administration of a dose for more than 30 min before or after the scheduled time of administration in the absence of an acceptable reason.	Wrong time error	The Right Time	Getting the Right Time Every Time!

#### Analyzing symptoms

(Incidence of medication errors)

##### Operational definition

See Methods section of this research paper. We would measure the Incidence of medication error by measuring the following rates through observation methods of process or self-reporting:

1. Wrong prescription error repetition rate
2. Wrong drug administration errors rate
3. Wrong dose administration errors rate
4. Wrong route administration errors rate
5. Wrong administration errors repetition rate

Operational definitions can be seen in Table 4.

**Table 4:** Operational definitions

Mission statement	Definition required for	Definition	Additional definitions
To reduce the medication error to less than 1 per 100 administrated medication doses	Medication errors	A medication error is any preventable event that may cause or lead to inappropriate medication use or patient harm while the medication is in the control of the health care professional, patient, or consumer. Medication errors are typically viewed as related to administration of a medication, but they can also include errors in ordering or delivering medication. The medication dose must actually reach the patient. If the incorrect dose is discovered and corrected before administration to the patient, no error occurs.	Prescribing error: it includes mistakes made by the physician when ordering a medication; incorrect drug selection, route, the frequent of administration,, dosage form, or instructions for use of a drug product. Dispensing error: The deviations from the physician’s order, made by staff in the pharmacy when distributing medications to nursing units or to patients in an ambulatory setting. Administration error: The deviating from the physician’s order as written in the patient’s chart. Monitoring error: it includes the failure to review a prescribed therapeutic plan for appropriateness and detection of problems, or weakness to use appropriate clinical or laboratory data for adequate assessment of patient response to prescribed therapy.

##### Define the boundaries

**Fig. 2:**
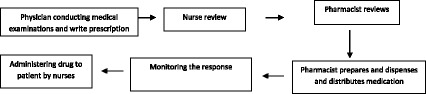
High level flow diagram in the medication use process

**Fig. 3:**
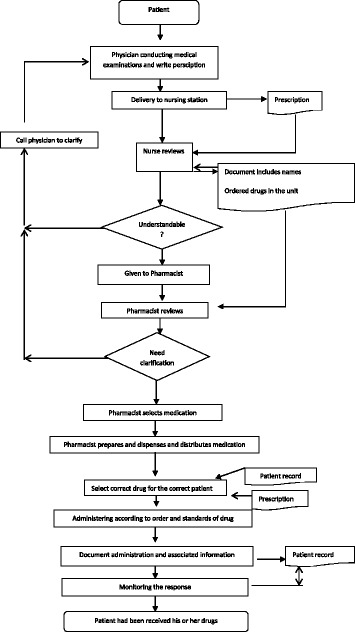
Detailed flow diagram medication use process

**Fig. 4:**
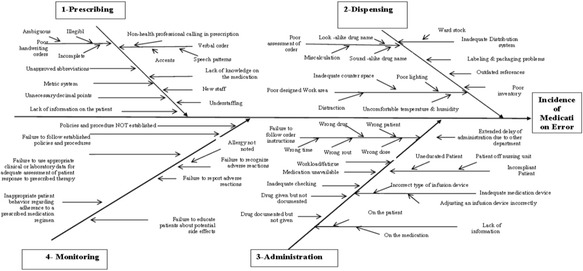
Cause-effect diagrams

Table 5 presents the boundaries of the process. Figure 2 shows the high level flow diagram in the medications use process, Fig. 3 shows detailed flow diagram of medication use process, and Fig. 4 shows cause effects diagrams.

**Table 5:** The boundaries of processes

Processes	Goals	Deficiencies
Prescribing	Assessing the need for and select the right drug .individuals the therapeutic regimen	- Illegibility - Abbreviations - Improper Dosing - Dosing Errors - Ordering medications to which patient was allergic - Duplicate therapy - Unclear/incomplete medication history
Dispensing	Preparing the drug and providing it in timely manner	- Labeling errors during repackaging - Lack of access to the right medication at the right time - Less control over inventory - Poor/no audit trail
Administration	Providing the right medication to the right patient when indicated	- Wrong patient - Wrong medication - Wrong time - Wrong dose - Wrong route
Monitoring	Monitoring of response and adverse events and evaluating of drug selection and regimen frequency and duration.	- Failure to recognize adverse reactions - Failure to report adverse reactions - Failure to educate patients about potential side effects

### Analysis phase

#### Formulate theories through brainstorming

This was used to determine the full range of possible causes. Table 6 shows the formulation of theories through brainstorming.

**Table 6:** Formulate theories through brainstorming

1. Abbreviations	31. Labeling (hospital’s)
2. Blanket orders	32. Leading zero missing
3. Brand names look alike	33. Measuring device inaccurate/inappropriate
4. Brand names sound alike	34. Monitoring inadequate/lacking
5. Brand/generic names look alike 6. Brand/generic names sound alike	35. Non-formulary drug 36. Non-metric units used
7. Calculation error	37. Packaging/container Design
8. Communication	38. Patient identification failure
9. Contraindicated, drug allergy	39. Preprinted order form
10. Contraindicated, drug/ drug 11. Contraindicated, drug/ food	40. Performance (human) deficit 41. Procedure/Protocol not followed
12. Contraindicated in disease	42. Pump, failure/malfunction
13. Contraindicated in pregnancy/breastfeeding	43. Pump, improper use
14. Decimal point	44. Reconciliation-admission
15. Diluents wrong	45. Reconciliation-discharge
16. Dispensing device involved	46. Reconciliation-transition
17. Documentation inaccurate/lacking	47. Reference material confusing/inaccurate
18. Dosage form confusion	48. Repackaging by hospital
19. Drug distribution system	49. Repackaging by other facility
20. Drug shortage	50. Similar packaging/labeling
21. Equipment design confusing/inadequate	51. Similar products
22. Equipment (not pumps) failure/malfunction	52. Storage proximity
23. Generic names look alike	53. System safeguards inadequate
24. Generic names sound alike	54. Transcription inaccurate /omitted
25. Handwriting illegible/ unclear	55. Unlabeled syringe/container
26. Incorrect medication activation	56. Verbal order confusing/incomplete
27. Information management system	57. Weight missing/inaccurate
28. Knowledge deficit/training Insufficient	58. Written order confusing/incomplete
29. Label (manufacturer’s) design	59. Workflow disruption
30. Label (hospital’s) design	

#### Cause-effect diagrams

We categorized the causes according to process steps

1. Prescribing
2. Dispensing
3. Administrating
4. Monitoring

#### Data collection

The data which we need are not available; we don’t have reports about the medications error and no registration system of any related data, so the method of data collection we selected was medication error causes- data sheet which must be filled by nurses. Table 7 shows the Medication Error Causes- Data Sheet.

**Table 7:** Medication Error Causes- Data Sheet. The purpose of this sheet is to investigate Why Medication Errors Occur? The following statements are all possible causes of medication errors. Please read them carefully, and indicate your answer using √ for the appropriate statements (please select only THREE statements)

I)-Causes Related Prescribing Phase		III)-Causes Related Administration Phase	
a. New staff	□	p. Failure to follow order instructions □ Wrong drug □ Wrong Patient □ Wrong time □ Wrong rout □ Wrong dose	□
b. Poor handwriting orders □ Ambiguous □ Illegible □ Incomplete	□		
c. Understaffing	□	q. Workload/fatigue	□
d. Unapproved abbreviations	□	r. Medication unavailable	□
e. Verbal order □ Non-health professional calling in prescription □ Speech patterns □ Accents	□	s. Inadequate checking	□
		t. Extended delay of administration due to other department (Lab levels, x-ray,.)	□
		u. Incompliant Patient □ Uneducated Patient □ Patient off nursing unit	□
f. Metric system	□		
g. Lack of knowledge on the medication	□		
		v. Inadequate medication device □ Incorrect type of infusion device □ Adjusting an infusion device incorrectly	□
h. Unnecessary decimal points	□		
i. Lack of information on the patient	□		
		w. Lack of information □ On the patient □ On medication	□
II)-Causes Related Dispensing Phase			
		x. Drug given but not documented	□
j. Poor assessment of order □ Look -alike drug name □ Sound -alike drug name □ Miscalculation	□	y. Drug documented but not given	□
		IV)-Causes Related Monitoring Phase	□
		z. Failure to recognize adverse reactions □ Allergy not noted	□
k. Poor designed Work area □ Inadequate counter space □ Poor lighting □ Distraction □ Uncomfortable temperature & humidity	□	aa. Failure to report adverse reactions	□
		bb. Failure to educate patients about potential side effects	□
		cc. Failure to use appropriate clinical or laboratory data for adequate assessment of patient response to prescribed therapy	□
l. Inadequate Distribution system □ Ward stock	□	dd. Inappropriate patient behavior regarding adherence to a prescribed medication regimen	□
		ee. Failure to follow established policies and procedures	□
m. Labeling & packaging problems	□		
n. Outdated references	□	ff. Policies and procedure NOT established	□
o. Poor inventory arrangement	□		

Note:

**Whereas nurses play multifarious roles in medication use process,** they are the cornerstone in the medication use process; nurses involve in all process steps and understand the process. They receive orders from physicians, receive drugs from pharmacy, administrate the drugs to the patient and finally monitor the response.

Therefore, Data sheet was set to investigate **“Why Medication Errors occur”, and** every nurse should select three causes of the sheet. One hundred and six sheets were filled and analyzed. The following table; Table 8, shows the total score of each cause of medication error.

**Table 8:** The total score of each cause of medication error

	Score^a^
I)-Causes Related Prescribing Phase	
a. New staff	9
b. Poor handwriting orders	24
c. Verbal order	14
d. Unapproved abbreviations	22
e. Understaffing	14
f. Metric system	19
g. Lack of knowledge on the medication	7
h. Unnecessary decimal points	16
i. Lack of information on the patient	11
II)-Causes Related Dispensing Phase	
j. Poor assessment of order	12
k. Outdated references	3
l. Labeling & packaging problems	8
m. Poor designed Work area	9
n. Inadequate Distribution system	9
o. Poor inventory arrangement	4
III)-Causes Related Administration Phase	
p. Failure to follow order instructions	8
q. Workload/fatigue	15
r. Medication unavailable	10
s. Inadequate checking	6
t. Extended delay of administration due to other department (Lab levels, x-ray,.)	8
u. Incompliant Patient	11
v. Inadequate medication device	4
w. Lack of information	11
x. Drug given but not documented	13
y. Drug documented but not given	1
IV)-Causes Related Monitoring Phase	
z. Failure to recognize adverse reactions	5
aa. Failure to report adverse reactions	4
bb. Failure to educate patients about potential side effects	12
cc. Failure to use appropriate clinical or laboratory data for adequate assessment of patient response to prescribed therapy	2
dd. Inappropriate patient behavior regarding adherence to a prescribed medication regimen	13
ee. Failure to follow established policies and procedures	15
ff. Policies and procedure NOT established	7

^a^Scores were calculated depending on nurses answers and notes they filled in Medication Error Causes- Data Sheet presented previousely in Table 7

#### Data-analysis

**Fig. 5:**
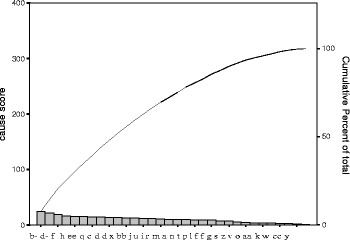
Pareto diagram- causes of medication error (*N* = 318) * \ Caption: Results from Table 9 (Pareto table --causes of medication error) were presented as Pareto Diagram

**Fig. 6:**
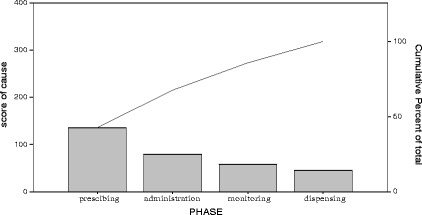
Pareto diagram -cause of error during phases of medication process (*N* = 318)

**Fig. 7:**
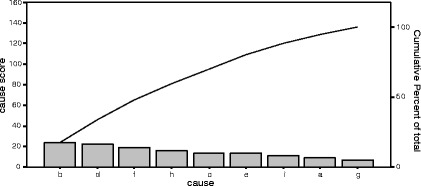
Pareto diagram- causes of medication error related prescribing phase (*N* = 318)

**Fig. 8:**
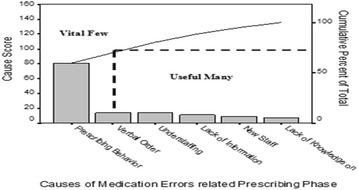
Pareto diagram- causes of medication error related prescribing phase after groping the causes related to prescribing behavior of physicians (poor handwriting orders, unapproved abbreviations, metric & apothecary systems and unnecessary decimal points) (*N* = 318)

Data-analysis tool is Pareto diagram, to focus on the vital few. The goal of the Pareto is to separate the causes of problems into the vital few and the useful many. Pareto diagram was used to present the results (Tables 9, 10, 11 and 12 & Figs. 5, 6, 7 and 8) reveal the contributors, magnitude and cumulative percent.

**Table 9:** Pareto table --causes of medication error (*N* = 318)

Cause of medication error	Score	Percent	Cumulative percent
b. Poor handwriting orders	24	7.5	7.5
d. Unapproved abbreviations	22	6.9	14.5
f. Metric system	19	6.0	20.4
h. Unnecessary decimal points	16	5.0	25.5
ee. Failure to follow established policies and procedures	15	4.7	30.2
q. Workload/fatigue	15	4.7	34.9
c. Verbal order	14	4.4	39.3
e. Understaffing	14	4.4	43.7
dd. Inappropriate patient behavior regarding adherence to a prescribed medication regimen	13	4.1	47.8
x. Drug given but not documented	13	4.1	51.9
bb. Failure to educate patients about potential side effects	12	3.8	55.7
j. Poor assessment of order	12	3.8	59.4
u. Incompliant Patient	11	3.5	62.9
i. Lack of information on the patient	11	3.5	66.4
r. Medication unavailable	10	3.1	69.5
m. Poor designed Work area	9	2.8	72.3
a. New staff	9	2.8	75.2
n. Inadequate Distribution system	9	2.8	78.0
t. Extended delay of administration due to other department (Lab levels, x-ray,.)	8	2.5	80.5
p. Failure to follow order instructions	8	2.5	83.0
l. Labeling & packaging problems	8	2.5	85.5
ff. Policies and procedure NOT established	7	2.2	87.7
g. Lack of knowledge on the medication	7	2.2	89.9
s. Inadequate checking	6	1.9	91.8
z. Failure to recognize adverse reactions	5	1.9	93.4
v. Inadequate medication device	4	1.3	95.7
o. Poor inventory arrangement	4	1.3	95.9
aa. Failure to report adverse reactions	4	1.3	97.2
k. Outdated references	3	0.9	98.1
w. Lack of information	3	0.9	99.1
cc. Failure to use appropriate clinical or laboratory data for adequate assessment of patient response to prescribed therapy	2	0.6	99.7
y. Drug documented but not given	1	0.3	100
Total	318	100

**Table 10:** Pareto table -cause of error during phases of medication process (*N* = 318)

Causes	Score	Percent	Cumulative percent
Causes Related Prescribing Phase	136	42.8	42.8
Causes Related Administration Phase	79	24.8	67.7
Causes Related Monitoring Phase	58	18.2	85.8
Causes Related Dispensing Phase	45	14.2	100
Total	318	100

**Table 11:** Pareto Table- causes of medication error related prescribing phase (*N* = 318)

Cause of medication error	Score	Percent	Cumulative percent
b. Poor handwriting orders	24	17.6	17.6
d. Unapproved abbreviations	22	16.2	33.8
f. Metric system	19	14	47.8
h. Unnecessary decimal points	16	11.8	59.6
c. Verbal order	14	10.3	69.9
e. Understaffing	14	10.3	80.1
i. Lack of information on the patient	11	8.1	88.2
a. New staff	9	6.6	94.9
g. Lack of knowledge on the medication	7	5.1	100
Total	136	100

**Table 12:** Pareto table

Cause of medication error	Score	Percent	Cumulative percent
Prescribing behavior of physicians	81	59.6	59.6
Verbal order	14	10.3	69.9
Understaffing	14	10.3	80.1
Lack of information on the patient	11	8.1	88.2
New staff	9	6.6	94.9
Lack of knowledge on the medication	7	5.1	100
Total	136	100	

Causes of medication error related prescribing phase after groping the causes related to prescribing behavior of physicians (poor handwriting orders, unapproved abbreviations, metric & apothecary systems and unnecessary decimal points) (*N* = 318)

##### Causes of medication error

Pareto diagram does not produce a clear picture of the vital few because each of the categories is nearly equal in the score. The data indicate no clear distinction among the categories.

All the bars on a Pareto diagram roughly have the same height, and it takes more than half of the categories to account for more than 60% of the quality effect.

Data were stratified by process steps, then we selected the first phase which had the higher scores and then we grouped the causes resulted from (poor handwriting orders, unapproved abbreviations, metric & apothecary systems and unnecessary decimal points).

#### Identify root causes

Root Causes are causes resulted from traditional prescribing behavior of physicians:

1. Poor handwriting orders

- Ambiguous
- Illegible
- Incomplete

2. Unapproved abbreviations
3. Metric systems
4. Unnecessary decimal points

The proposed root causes are controllable because they are related to one factor of the process applied by the physicians who prescribe the drugs (prescribing behavior or practice).

### Improvement phase

#### Evaluate the alternatives

##### Formulate remedies through brainstorming

1. **Automation and technology:** In hospitals, this can be by the presence of computerized medication records such as electronic prescribing, bar coding, and automated drug-dispensing systems.
2. **Demand on the nursing staff:** Strategies to promote greater accuracy in drug administration account for increased demand on licensed nurses.
3. **Education & training of nursing staff:** Many hospitals spend a lot of money on high-technology equipment, but not enough on educating the nurses. This remedy is very important as nurses are who administrate the medications in most cases.
4. **Standardized general principle & practices of medication administration through six rights:** Nurses attempt to ensure that the Right **drug** is given in the Right **dose** at the Right **time** via the Right **route** to the Right **patient** and with right **documentation**
5. **Double check system:** This is by Double-check for every medication every time by a second person.
6. **Organization’s Policies & Procedures:** They are less expensive strategies that can be applied in the hospitals to reduce medication administration errors.
7. **Suitable work environment:** Suitable work environments should be available for the safe preparation of drugs
8. **Reporting about the incidence of medication error:** Reporting about the drug administration errors via incidence report is considered a professional and ethical responsibility of all health care providers
9. **No punitive actions:** Managers are responsible for ensuring that nurses and other providers are punished for the medication errors they make. Hence, error reporting is encouraged.
10. **Empowerment:** Nurses’ participation in problem solving is the best way to derive strategies that will be effective and feasible. Nurses have to be involved from their unit level through the hospital policy level in decisions affecting medication administration accuracy
11. **Medication Safety Committee:** Every hospital should have a medication safety committee

For evolution purpose we have organized the remedies in two main strategies that include the following:

- **Strategy (I):** Improvement of handwritten prescriptions.
- **Strategy (II):** Eliminating all handwritten prescriptions by Implementing computerized order entry

##### Evaluate the alternatives

Strategies to Improve Medication Safety:

1. Improve handwritten prescriptions by Support efforts to increase prescription legibility and Developing & disseminating guideline to improve handwritten prescriptions
  - Use of standard prescription preparation practices in the education and continuous education of physicians.
  - Standardize prescription writing way and its rules, include the purpose(guideline)
  - Establish and use the standard terminology elements.
  - Encourage physicians to avoid using drug name abbreviations on all prescriptions and drug orders.
  - Ask the physicians to add a notation of purpose (not necessarily diagnosis) on all prescriptions.
  - Ask the physicians to add a notation on the prescription for a child patient, and to mention the exact age of the child who is less than 14 years of age.
2. Eliminate all handwritten prescriptions.
  - Use electronic entry, hand-held computer, or other similar technology
  - Use automated drug-ordering systems.
  - Implement physician order entry.
  - Ask for Physicians’ direct computer entry of prescriptions
  - Physician entry of prescriptions on a computer reduces transcription errors and shows potentially problematic prescriptions. For instance, it shows an improper dose being prescribed or a drug that might interact with another medication taken by the patient.
  - Automated hospital dispensing systems notify nurses when a drug is to be administered. The systems also record what has been given and when as well as reduce the delays in giving patients their medications and decrease other administration errors.
  - Barcoding hospital medications: Machine-readable labels can facilitate matching patients with their prescribed medications and documenting drug dispensing and administration.
  - Computerized medication errors monitoring: Computer programs are designed to show potential medication errors, using data from electronic patient medical records (e.g., orders for known antidotes or specific laboratory test abnormalities).

**Evaluation criteria:** The two aforementioned strategies were evaluated according to the following criteria:

**Table:** 

- Total Cost	- Implementation Duration
- Impact on the Problem	- Uncertainty about Effectiveness
- Benefit/Cost Relationship	- Health & Safety
- Cultural Impact/Resistance to Change	- Environment

Each strategy was given a score from H to L through M where (H = High Desirability and M = Medium Desirability, while L = Low Desirability). Table 13 shows Remedy Selection Matrix and according to this matrix the selected strategy was **Improvement of handwritten prescriptions** by supporting traditional process of prescribing.

**Table 13:** Remedy selection matrix

Criterion	Remedy 1	Remedy 2
Remedy name	Improve handwritten prescriptions	Eliminate all handwritten prescriptions
Total cost	M	L
Impact on the problem	M	H
Benefit/Cost relationship	H	M
Cultural impact/resistance to change	L	M
Implementation time	M	L
Uncertainty about effectiveness	M	L
Health & safety	H	M
Environment	M	L
Summery (Rate 1 for best, 2 for next, and so on.)	1.9	2.4

*H* high desirability, *M* medium desirability, *L* low desirability

**Fig. 9:**
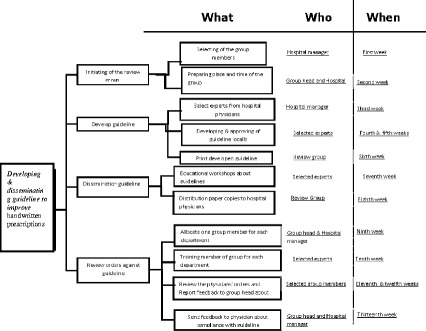
Planning matrix

- The planning matrix, Fig. 9, shows the process that will be conducted to implement the selected remedial strategy.

Planning step included:

- Establishing review group (committee)

- Developing guideline for prescribing

- Disseminating guideline to all physicians through educational workshops

- Training members of review group to review prescribing orders in accordance with the guideline recommendations **Design remedy:** The required resources:

- *People:* a review group to be consisted of a qualified physician as head of the group, a nurse assistant to the physician, five secretaries for hospital units and departments, and one clerk. Unit secretary in each department should be trained to review drug orders (prescriptions) in accordance with the guideline recommendations, and report to group administrators.
- *Money:* Costs of development and dissemination of the guidelines, training of medical secretary
- *Duration:* nine weeks
- *Materials:* Place of review group and material needed for print the guideline and circulation of the guideline to all the physicians in the hospital

#### Design for culture

- Sources of barriers and aid.- Countermeasures needed to overcome barriers.

**Barriers are**: Shortage in nurses, Physician acceptance, Physician time.

**Aids are**: Involvement & commitment of Top Management

**Counter measures include** Training of medical secretary, Participating in developing the guideline, Educational workshops about guidelines.

**Fig. 10:**
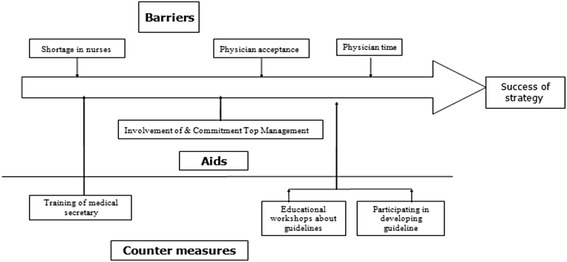
The design of culture

The design for culture can be seen in Fig. 10

#### Prove effectiveness: (pilot test, implement plan)

- Pilot test

The strategy was implemented on the outpatients on a limited scale

- Implement Plan

1. Educational workshops for physicians about Guideline Recommendations
2. Training of unit secretaries on their responsibilities which include review prescribing orders in accordance with the Guideline Recommendations (Table 14).

**Table 14:** Guideline recommendations to improve handwritten prescriptions

Guideline Recommendations to Improve Handwritten Prescriptions
1. Always write legibly.
2. Provide complete information with orders and prescriptions, e.g., patient’s full name, date of birth, weight if appropriate.
3. Do not use abbreviations for drug names.
4. Provide clear, unambiguous, and complete directions for use.
5. Do not use abbreviations for use that can be confused.
6. Use the metric system only.
7. Do not use trailing zeros (1.0 g).
8. Always use a zero before a decimal point (0.1 mg).
9. Spell out “units”; never use abbreviation “U”.
10. Do not use “μg” to abbreviate micrograms.
11. Always provide dosing equation, patient weight or body surface area, and calculated doses for chemotherapies and pediatric patients.
12. Provide indication for medication use with prescriptions.
13. Use verbal orders only when necessary. Have the receiving person read the order back. Spell out potential sound and look alike drugs.
14. Always write complete orders.
15. Always write out all orders; do not write orders such as “resume pre-op meds”.

### Control phase

#### Preservation the remedy

**Fig. 11:**
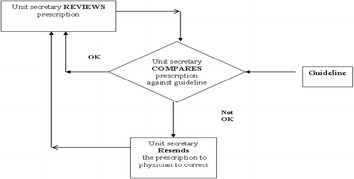
Feedback loop

**Fig. 12:**
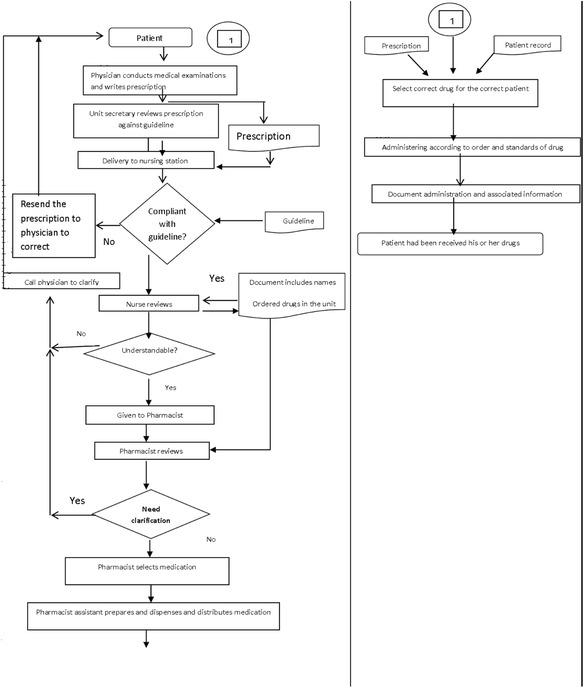
Detailed flow diagram new medication use process

Unit secretary reviews prescriptions in accordance with the guideline, if order is: ambiguous, illegible or incomplete or includes unapproved abbreviations. Then the unit secretary resends the prescription to physician to correct it. Figure 11 shows the feedback loop, and Fig. 12 shows Detailed flow diagram new medication use process.

#### Audit the controllers

○ The Head of review group should aggregate the rate of incidence of medication errors and should report the percentage to the hospital manager regularly.

○ The Head of review group should count the percentage of compliance of the physicians with the guideline and should send to the hospital manager regular reports about the level of compliance of the physicians. Then, the hospital manager will handle this issue with the physicians in his regular meetings with the medical staff.

○ Each prescription should be reviewed in dependence on the **Review Sheet** in accordance with the **Guideline Recommendations (1),** Additional file 1

○ The data that were included in the Review Sheet in accordance with the Guideline Recommendations should be aggregated **in Daily Review Sheet (2),** Additional file 2

○ For each physician the level of compliance with the guideline should be assessed using the formulas of **Indicators for Guideline (3),** Table 15

**Table 15:** Indicators for guideline (3)

Numbers of illegible orders in the specific period	X100
Number of all orders in the same specific period	
Numbers of incomplete orders in the specific period	X100
Number of all orders in the same specific period	
Numbers of orders which included abbreviations for the specific period	X100
Number of all orders in the same specific period	
Numbers of illegible orders for specific physician in the specific period	X100
Number of all orders for specific physician in the same specific period	
Numbers of incomplete orders for specific physician in the specific period	X100
Number of all orders for specific physician in the same specific period	
Numbers of orders which included abbreviations for specific physician the specific period	X100
Number of all orders for the specific physician in the same specific period	

○ Results of computing the indicators should be compared with the standards. Each **division should** report to the manager to take the appropriate **corrective actions**.

## Conclusions

Since medication errors are a global threat for healthcare workers’ and patients’ safety we tried in our study to apply Six Sigma set of steps (DMAIC) integrated into TQM tools to recommend a new technique to prevent medication error incidences in healthcare sections. First, we defined the medication errors and determined their problems to set the objective of this study which is reducing the incidence of administrated medication doses to meet the global standards without any extra cost. Then, we moved to the “measure” phase of six sigma approach. We were able to determine SIPOC for medication process, listen to The Voice Of The Customer, and to define the operation with its boundaries. In analyzing step, we formulated theories through brainstorming to consider the full range of possible causes of medication error incidences by data collection using **Medication Error Causes- Data Sheet**. Then, we analyzed the collected data using Pareto Diagrams to determine the Vital Few. In this step, we found that Prescribing Error Incidences occur in 42.8% after which come Administrating Errors, monitoring Errors and Dispensing Errors, with 24.8%,18.2%,14.2% respectively. In prescribing error incidences the poor handwriting orders had a higher score than other sub-causes. Therefore, we compared between two strategies for this sub-causes using **Remedy Selection Matrix** in the “Improve” phase of six sigma steps. The two main improvement strategies were either to improve the prescription hand writing or to eliminate them by automation. According to this matrix, the selected choice was to support handwritten prescriptions by suggestion of assistant sheets; **Guideline Recommendations to Improve Handwritten Prescriptions** to be used by the physicians working in the Hospital. To prevent the remedy, we kept the feedback loops as short as possible. A number of sheets were also suggested in the control step to audit the controllers. These sheets are **Review Sheets** in accordance with the **Guideline Recommendation**, **Daily Review Sheets**, **and Indicators for Guideline**. Finally, we hope that this proposed strategy for improving mediation use will be applied by other healthcare researches, to know how much effective it is and how much it does improve the medication use process which ensures that each patient will have his or her own suitable drugs away from medication errors and the adverse effect incidences.

## Additional files

Additional file 1: Table S1. Review Sheet against Guideline Recommendations (1) (N= 318). (DOCX 14 kb)

Additional file 2: Table S2. Daily Review Sheet (2). (DOCX 13 kb)

## Abbreviations

CCRs: Critical Customer Requirements
CDS: Controlled Drug Substances
CTQs: Critical to Quality
DDIs: Drug-Drug Interactions
DMAIC: Define, Measure, Analyze, Implement, Control
TQM: Total Quality Management
