# Supplementary material for: Using total quality management approach to improve patient safety by preventing medication error incidences**
Source: BMC Health Serv Res. 2017 Sep 4;17:621. doi: 10.1186/s12913-017-2531-6 (PMC5584345; doi:10.1186/s12913-017-2531-6)
Supplement: Supplementary file 1 — Review Sheet against Guideline Recommendations (1) (N= 318). (DOCX 14 kb) [file 12913_2017_2531_MOESM1_ESM.docx]

**Additional file 1: Table S1 Review Sheet against Guideline Recommendations (1) (N= 318).**

| **Percentage** | **Number** | **No** | **Yes** | **Guideline standards** |
| --- | --- | --- | --- | --- |
|  |  | 5 | 5 | 1. **Order is legible.** |
|  |  | 1. **providing complete information** | | |
|  |  | 5 | 5 | - patient’s full name, |
|  |  | 5 | 5 | - date of birth, |
|  |  | 5 | 5 | - weight if appropriate, |
|  |  | 5 | 5 | 1. **Use abbreviations for drug names.** |
|  |  | 1. **Providing directions for use** | | |
|  |  | 5 | 5 | - clear, |
|  |  | 5 | 5 | - unambiguous, |
|  |  | 5 | 5 | - Complete |
|  |  | 5 | 5 | 1. **Use confused (unapproved ) abbreviations** |
|  |  | 5 | 5 | 1. **Use the metric system** |
|  |  | 5 | 5 | 1. **Use trailing zeros (1.0 gm).** |
|  |  | 5 | 5 | 1. **Use a zero before a decimal point (0.1 mg).** |
|  |  | 5 | 5 | 1. **Use abbreviation “U”.** |
|  |  | 5 | 5 | 1. **Use “ug” to abbreviate micrograms.** |
|  |  | 1. **Providing for pediatric patients** | | |
|  |  | 5 | 5 | - dosing equation, |
|  |  | 5 | 5 | - patient weight |
|  |  | 5 | 5 | - body surface area, |
|  |  | 5 | 5 | 1. **Providing calculated doses for chemotherapies patients** |
|  |  | 5 | 5 | 1. **Providing indication for medication use with prescriptions.** |
|  |  | 5 | 5 | 1. **Use verbal orders** |
|  |  | 5 | 5 | 1. **Writing complete orders.** |
|  |  | 5 | 5 | 1. **Writing incomplete orders such as “resume pre-op meds”.** |

| Name of doctor:  Date:  Hospital Department or Outpatient Clinic:  Patient name:  Reviewer name: |
| --- |
