# Supplementary material for: Using total quality management approach to improve patient safety by preventing medication error incidences**
Source: BMC Health Serv Res. 2017 Sep 4;17:621. doi: 10.1186/s12913-017-2531-6 (PMC5584345; doi:10.1186/s12913-017-2531-6)
Supplement: Supplementary file 2 — Daily Review Sheet (2). (DOCX 13 kb) [file 12913_2017_2531_MOESM2_ESM.docx]

**Additional file 2: Table S2 Daily Review Sheet (2)**

| **Physician Name** | Ordering Date | | | | | | | Abbreviation | | | Incomplete | | Illegible | | **Reviewer name** |
| --- | --- | --- | --- | --- | --- | --- | --- | --- | --- | --- | --- | --- | --- | --- | --- |
|  | Year | Moth | | Day | | Time | | No | | Yes | No | Yes | No | Yes |  |
|  |  | |  |  |  | |  | |  | |  |  |  |  |  |
|  |  | |  |  |  | |  | |  | |  |  |  |  |  |
|  |  | |  |  |  | |  | |  | |  |  |  |  |  |
|  |  | |  |  |  | |  | |  | |  |  |  |  |  |
|  |  | |  |  |  | |  | |  | |  |  |  |  |  |
|  |  | |  |  |  | |  | |  | |  |  |  |  |  |
|  |  | |  |  |  | |  | |  | |  |  |  |  |  |
|  |  | |  |  |  | |  | |  | |  |  |  |  |  |
|  |  | |  |  |  | |  | |  | |  |  |  |  |  |
|  |  | |  |  |  | |  | |  | |  |  |  |  |  |
|  |  | |  |  |  | |  | |  | |  |  |  |  |  |
|  |  | |  |  |  | |  | |  | |  |  |  |  |  |
|  |  | |  |  |  | |  | |  | |  |  |  |  |  |
|  |  | |  |  |  | |  | |  | |  |  |  |  |  |
|  |  | |  |  |  | |  | |  | |  |  |  |  |  |
|  |  | |  |  |  | |  | |  | |  |  |  |  |  |
|  |  | |  |  |  | |  | |  | |  |  |  |  |  |
|  |  | |  |  |  | |  | |  | |  |  |  |  |  |
|  |  | |  |  |  | |  | |  | |  |  |  |  |  |
|  |  | |  |  |  | |  | |  | |  |  |  |  |  |
|  |  | |  |  |  | |  | |  | |  |  |  |  |  |
|  |  | |  |  |  | |  | |  | |  |  |  |  |  |
|  |  | |  |  |  | |  | |  | |  |  |  |  |  |
|  |  | |  |  |  | |  | |  | |  |  |  |  |  |
|  |  | |  |  |  | |  | |  | |  |  |  |  |  |
|  |  | |  |  |  | |  | |  | |  |  |  |  |  |
|  |  | |  |  |  | |  | |  | |  |  |  |  |  |
|  |  | |  |  |  | |  | |  | |  |  |  |  |  |
|  |  | |  |  |  | |  | |  | |  |  |  |  |  |
|  |  | |  |  |  | |  | |  | |  |  |  |  |  |
|  |  | |  |  |  | |  | |  | |  |  |  |  |  |
|  |  | |  |  |  | |  | |  | |  |  |  |  |  |
|  |  | |  |  |  | |  | |  | |  |  |  |  |  |
|  |  | |  |  |  | |  | |  | |  |  |  |  |  |
|  |  | |  |  |  | |  | |  | |  |  |  |  |  |
|  |  | |  |  |  | |  | |  | |  |  |  |  |  |
|  |  | |  |  |  | |  | |  | |  |  |  |  |  |
|  |  | |  |  |  | |  | |  | |  |  |  |  |  |
